# For the Love of Nature: Exploring the Importance of Species Diversity and Micro-Variables Associated with Favorite Outdoor Places

**DOI:** 10.3389/fpsyg.2017.02094

**Published:** 2017-12-01

**Authors:** Morgan F. Schebella, Delene Weber, Kiera Lindsey, Christopher B. Daniels

**Affiliations:** ^1^Natural and Built Environments Research Centre, University of South Australia, Adelaide, SA, Australia; ^2^Barbara Hardy Institute, University of South Australia, Adelaide, SA, Australia; ^3^Hawke Research Institute, University of South Australia, Adelaide, SA, Australia; ^4^School of Pharmaceutical and Medical Sciences, University of South Australia, Adelaide, SA, Australia

**Keywords:** biodiversity, ecological literacy, favorite places, nature connectedness, restorative environments

## Abstract

Although the restorative benefits of nature are widely acknowledged, there is a limited understanding of the attributes of natural environments that are fundamental to restorative experiences. Faced with growing human populations and a greater awareness of the wellbeing benefits natural environments provide, park agencies and planners are increasingly challenged with balancing human and ecological outcomes in natural areas. This study examines the physical and experiential qualities of natural environments people referred to when describing their connection to their most valued natural environments in an online questionnaire. Recruited primarily via a public radio program, respondents were asked to identify their favorite places and explain what they loved about those places. Favorite places are considered exemplars of restorative environments and were classified based on an existing park typology. Reasons people liked particular sites were classified into three domains: setting, activity, or benefit. Content analysis was used to identify the attributes most commonly associated with favorite places. These attributes were then related to the four components of restorative environments according to Attention Restoration Theory. In contrast to previous research, we found that “fascination” was the most important component of favorite places. Possible reasons for this contrast, namely, respondents' median age, and the likelihood of a high degree of ecological literacy amongst the study population are discussed. South Australians' favorite environments comprise primarily hilly, wooded nature parks, and botanical gardens, in stark contrast to the vast arid areas that dominate the state. Micro-variables such as birds, plants, wildlife, native species, and biodiversity appear particularly important elements used to explain people's love of these sites. We discuss the implications of these findings and their potential value as an anchor for marketing campaigns seeking to encourage contact with nature, as well as education programs designed to improve people's understanding of important but intangible concepts such as biodiversity. The findings have clear, practical implications for park managers given the modifiable nature of many of the attributes identified as being most important to our respondents, and we believe attention to such elements has the potential to simultaneously enhance people's nature experiences, optimize restorative outcomes, and improve environmental stewardship.

## Introduction

For many people contact with nature is no longer a by-product of everyday life. For the vast majority of human history, we relied upon the natural environment for food, water, and shelter in very direct and unambiguous ways, as all species do. Today, although we are equally dependent upon the environment to sustain us, the majority of the world's population now resides in towns and cities (The United Nations, 2014), where they are largely sheltered from the natural processes and ecosystem services that make their existence possible (Miller, 2005). The consequences of this separation between “people” and “nature” are two-fold. Firstly, as each succeeding generation becomes increasingly disconnected from the natural world, the collective importance placed upon the environment by urban populations is likely to diminish (Pyle, 2002). In turn, this may lead to reduced advocacy and funding for conservation and biodiversity protection, which has long-term global implications. Secondly, a lack of contact with the environment is thought to be a contributing factor to the increasingly poor health and wellbeing of urban inhabitants (Maller et al., 2008), which some scholars suggest is the result of a failure to fulfill our inherent biological need to spend time in nature (Wilson, 1984).

In order to develop policies and practices that see contact with nature become commonplace again, we must first understand the activities, environmental settings, and benefits that encourage people to seek out nature experiences. As exemplars of restorative, health-giving environments, we believe investigating perceptions of “favorite places” and the attributes people describe when explaining their connection to these settings will provide valuable information for urban planners seeking to optimize the health benefits of nature. In this paper we explore the attributes of outdoor environments that people place great personal importance on and consider the implications of these findings to modern societies.

### The psychological benefits of contact with nature

The well-being benefits of contact with nature has long been a topic of interest to researchers across diverse disciplines, and there is now a broad evidence base supporting a positive relationship between human health and nature (e.g., Velarde et al., 2007; Keniger et al., 2013). The influence of the quality of natural environments on mental health, and the relative importance of individual environmental variables on psychological outcomes, are examples of areas that remain poorly explored (Gascon et al., 2015). Despite concerns raised by researchers regarding the methodological limitations and lack of consistency in the results of some nature-health research (Lee and Maheswaran, 2011; Hartig et al., 2014; Gascon et al., 2015), studies into human interactions with nature are generally supportive of the premise that natural environments have a more favorable effect on human psychological health than do urban or built environments, whether experienced indirectly or directly through visual (Kaplan and Kaplan, 1989; Ulrich et al., 1991), auditory (Alvarsson et al., 2010; Annerstedt et al., 2013), or olfactory contact (Tsunetsugu et al., 2010). People living in urban areas with more green space are often found to have better mental health and perceived general health than people living in urban areas with less green space, even when controlling for a range of extraneous factors such as income and marital status (De Vries et al., 2003; Beyer et al., 2014). Studies suggest that visiting or viewing natural settings may improve concentration in children with ADHD (Taylor and Kuo, 2009); reduce anxiety in hospital patients (Beukeboom et al., 2012); minimize perceived pain and discomfort (Diette et al., 2003); restore cognitive function (Hartig et al., 1991); and facilitate recovery from stress (Ulrich et al., 1991; Beil and Hanes, 2013). Furthermore, contact with nature has been associated with lower frustration (Aspinall et al., 2015); increased happiness (Mackerron and Mourato, 2013); improved mood and self-esteem (Barton and Pretty, 2010); and faster recovery from surgery (Ulrich, 1984). For these reasons and more, it is not surprising that the presence of natural space in urban areas is considered by many to be a form of “upstream health promotion” that has the potential to positively influence human wellbeing on a population-wide scale (Maller et al., 2006).

In spite of a growing body of research documenting the importance of nature to human health, natural space in many urban areas is decreasing (McDonald et al., 2010; Sivam et al., 2012). The demand for infrastructure to meet the needs of growing urban populations is often met through the development and modification of natural areas. This is of great concern, as a lack of green space reduces opportunities to experience nature, and may be impacting the mental health of urban inhabitants (Bratman et al., 2015). In Australia, urban residents are more likely to suffer from high or very high psychological distress (Australian Bureau of Statistics, 2011) and face a higher risk of developing substance use disorders than rural residents (Cantwell et al., 2012). The story is similar in other western nations such as Great Britain, where people residing in cities have been found to have poorer mental health than their rural counterparts (Paykel et al., 2000). Some researchers have estimated that urban inhabitants face a 21% greater risk of developing an anxiety disorder, and a 39% greater risk of developing a mood disorder than rural inhabitants (Peen et al., 2010). The proportion of the global population residing in urban areas is expected to continue rising for decades to come (The United Nations, 2014), and with this shift, we can expect to see continued increases in psychological disorders such as chronic stress, anxiety, and depression. Providing planners with information about the attributes of natural environments that optimize recovery from stress and fatigue may therefore contribute to improving quality of life for many people.

### A healthy urban environment

Increasing the amount of natural space in urban areas, or “neighborhood greening” has been proposed as one possible approach to helping create a healthier living environment for urban inhabitants (Beyer et al., 2014). However, some studies indicate that simply having more green space in urban settings may be ineffective at improving human health (Richardson et al., 2012) or promoting greater use of natural environments (Francis et al., 2012). After decades of research that followed a largely dichotomous “urban” vs. “natural” environments approach, many scholars have noted there is a dearth of information regarding the actual attributes of natural environments that are required to facilitate psychological benefits (Frumkin, 2003; Nordh et al., 2009; Keniger et al., 2013). More recently, researchers have begun focusing on the “micro” features of natural settings that might promote greater use and improve mental health outcomes in urban areas. As opposed to “macro” features, which typically include landscape-scale elements such as the quantity of green space in a given area, or its proximity to people's homes, “micro” features refer to site-specific differences between green spaces that might encourage use or enhance visitor experiences. Such “micro” features may include particular vegetation types, opportunities to view wildlife, specific landscape elements such as creeks, and physical amenities such as trails or exercise equipment. In Perth, Western Australia, Francis et al. (2012) used the Public Open Space Tool (POST) to assess park quality, considering “micro” variables such as walking trails, shade, birdlife, and the presence of water as contributors to park quality, based on the opinion of an expert panel. Their study concluded that the quality of green space in one's neighborhood was more important to one's mental health than the quantity of green space or the frequency with which one visited it. Other studies have found that different types of urban green space facilitate different types of health benefits (Brown et al., 2014); that the psychological benefits of contact with nature may be positively influenced by biodiversity (Fuller et al., 2007; Carrus et al., 2015) or perceived biodiversity (Dallimer et al., 2012); and that restorative outcomes may be associated with naturalness in pocket parks (Nordh et al., 2009). In this study, we aimed to identify the types of natural environments and “micro” attributes that are most important to South Australians. Using self-reported “favorite places” as exemplars of restorative environments, we also explored the relative importance of the key components central to Attention Restoration Theory (ART) (Kaplan and Kaplan, 1989).

### Restorative experiences and favorite places

The restorative benefits of natural environments are widely documented (e.g., Kaplan and Kaplan, 1989; Hartig et al., 2003), and have been a prominent feature of nature-health research for decades. Restoration refers to the psychological and/or physiological recovery one experiences during exposure to certain environments, and is most commonly explained by two dominant theories in the field: ART and Stress Reduction Theory (SRT). The two theories attempt to describe the mechanisms by which natural environments have a positive effect on human wellbeing, with ART concerning recovery from cognitive or attentional fatigue (Kaplan and Kaplan, 1989), and SRT concerning recovery from stress (Ulrich, 1983). In nature-health research, ART and SRT have commonly been regarded as “complementary perspectives that focus on different aspects of the restorative process” (Joye and Van Den Berg, 2013, p. 59).

According to ART, an environment is more likely to be restorative if it exhibits four characteristics: (1) it allows for a feeling of “being away” by being geographically and/or psychologically distant from the daily hassles of life and the sources of attentional fatigue; (2) it has sufficient “extent” and scope to allow for an immersive experience; (3) it is able to offer “compatibility” with the intentions/needs of the person experiencing the environment; and (4) it provides “soft fascination” to catch one's attention without cognitive effort. As opposed to fascination in general, which may be derived from stimuli such as car accidents or violent scenes, settings that are rich in soft fascination—such as “the play of light on foliage” (Kaplan and Kaplan, 1989, p. 193)—capture our involuntary attention in a non-threatening way, allowing the mind to wander and our attentional capacity to replenish. Generally, these features are more characteristic of natural rather than urban or built environments (Kaplan and Kaplan, 1989).

Natural environments are frequently cited as being “favorite places” of participants in restoration and place attachment research (Newell, 1997; Korpela and Ylén, 2007). Favorite places are those locations that individuals have formed an “emotional tie or affective bond” with (Korpela et al., 2009, p. 96). They are places that one might “value being in more than any other place” (Korpela et al., 2001, p. 579), or would choose to protect from “damage or destruction” above all others (Newell, 1997, p. 500). It has been suggested that this emotional bond forms because favorite places facilitate self-regulation, the act of mentally processing the psychological influences of external factors such as emotionally arousing situations (Korpela and Ylén, 2007) or sensory stimuli (Korpela, 1992). Self-regulation occurs when one applies mental, social, physical, or environmental strategies to help regulate their feelings and maintain their sense of self, or self-esteem. As restorative experiences may involve “reflection on oneself and one's place in the world” (Korpela and Hartig, 1996, p. 222), interacting with natural environments can be considered a form of environmental self-regulation (Korpela and Ylén, 2007). People are known to actively seek out natural environments when they are in need of restoration (e.g., Irvine et al.'s, 2013) and preferences for nature are found to be higher in individuals who are in greater need of restorative experiences (Hartig and Staats, 2006). According to Korpela et al. (2001, p. 573) “places that a person can rely on for restorative experiences are thus more likely to be places for which attachments develop over time and that in turn come to figure in place identity.” Tellingly, individuals prescribed with visiting their favorite places have been shown to experience significantly stronger restorative outcomes than individuals visiting other places (Korpela et al., 2009). For this reason, favorite places have been used as a “window” into restorative environments in previous research (Korpela et al., 2008, p. 637).

### Favorite places and environmental attributes

As “exemplars” of environments used in self-regulation and restorative experiences (Korpela and Hartig, 1996), an evaluation of the types of natural environments people consider to be their favorite places is relevant, as is identifying the elements people use to explain why an area is favored. A search for “favorite place” or “favourite place” literature published in peer reviewed journals over the past 30 years revealed only 10 articles. The majority of these articles were conducted by Korpela and associates, and have been primarily based in Europe. Two studies took place in the United States (Newell, 1997; Korpela et al., 2001), one of which also collected data in Ireland and Senegal (Newell, 1997). No explicit “favorite place” research appears to have been conducted in Australia. Each article was assessed to identify the types of environments and environmental characteristics that respondents valued most highly (Table 1).

**Table 1:** Favorite place types and features identified in previous research.

**Author/s**	**Favorite Place Types**	**Method of identifying favorite places**	**Consideration of ART**	**Consideration of attributes**	**Study Population**
Korpela, 1989	Focus was on feelings created by the environment, rather than the physical characteristics of it. However, features frequently described by respondents included: homelike, peaceful, secure, comforting, huge, indomitable, powerful, beautiful, silent, rugged, pleasant smell of wood, and colorfulness and grayness and ugliness at the same time.	Survey and essay. Survey: the 9 and 12-year old students answered 10 verbally delivered questions, asking them to describe their favorite place and why they like to visit it. Essay: the 17-year old students were asked to write an essay about their favorite place, its features, the feelings it gives them, and the mood they are in when they visit it.	No	Yes	Students aged 9, 12, and 17 years old, in and near Tampere, Finland
Korpela, 1991	Most common types of favorite places were: private homes (39%), restaurants/downtown (16%), natural settings (14%), sport facilities (14%), clubs (7%), and “other,” such as a car or motorcycle (10%).	Essay: in first study, respondents were asked to write an essay on their favorite place, explaining why it was important to them and what experiences and feelings they had there. In second study, participants were asked to write an essay on their experiences in their favorite place, focusing on the situations and feelings that motivated them to go there.	No	No	Students aged 17–18 years, near Tampere, Finland
Korpela, 1992	Most common types of favorite places were: private homes (39%), natural settings (15%), restaurants/downtown (15%), sport facilities (13%), clubs (7%), and “other,” such as a car or motorcycle (7%).	Essay: students were asked to write an essay about their favorite place, describing what sort of place it was, why it was their favorite place, and what personally important thoughts, feelings or experiences they have had there.	No	No	Students aged 17–18 years, near Tampere, Finland
Korpela and Hartig, 1996	Using a list of characteristics, respondents indicated the extent to which they were present in their favorite places. The most frequently reported characteristics were: beautiful views (83.3%), sunlight (83.1%), the presence of water (73.1%); and the presence of personal belongings (56.4%).	Survey: respondents were asked to evaluate seven settings, including a “favorite” and “unpleasant” place in their life, by completing the Perceived Restorativeness Scale (PRS) and the Zuckerman Inventory of Personal Reactions (ZIPERS).	Yes	Yes	Students from the University of Tampere, Finland. Aged 19–46 years
Newell, 1997	Natural sites in general were the favorite places of respondents. Ten place type categories ranked according to frequency: (1/2) “personal bedroom/belongings” were equal with “outdoors, nature, the Earth”; (3) beaches/coastline; (4) family home and surroundings; (5) built environment, pubs, streets; (6/7) woods/forests were equal with countryside/fields; (8) mountains; (9) recreation areas/parks; (10) rivers, lakes, and ponds.	Survey: open-ended responses to a question about their one favorite place “to save from damage or destruction.”	No	No	Psychology and sociology students from universities in: USA, Ireland and Senegal. Aged 18–45 years
Korpela et al., 2001	Largest proportion of favorite places (48%) were natural spaces, followed by residential sites (19%); geographic areas such as a country or city (16%); leisure settings such as amusement parks and zoos (5%); and school/university settings (4%).	Survey: open-ended responses to questions about their favorite place. Respondents asked to imagine being in their favorite place, “that one place in which you have most enjoyed spending time, or that you have valued being in more than any other place. Perhaps you view this place as being particularly significant in your life.” Also completed the PRS.	Yes	No	Psychology students from Berkeley, United States. Aged 17–47 years
Korpela and Ylén, 2007	Natural sites favorite places of 51% of respondents. No figures provided for place types, but the most frequently mentioned favorite natural places were nearby parks, woods, and seashores.	Survey: open-ended responses to the question: “Think about your residential area for a moment. What is your *favorite* place within the area? This place may be located indoors or outdoors.”	No	No	Residents of four residential areas in Helsinki, Finland. Average age 40 years
Korpela et al., 2008	Natural sites favorite places of majority of respondents. Sixteen favorite place types were grouped into five main place types. Ranked according to frequency: (1) extensively managed natural areas, e.g. woods, forests, meadows; (2) built-up green spaces, e.g. parks; (3) waterside environments, e.g. beaches and harbors; (4) exercise and activity/hobby areas, e.g., playgrounds and sports ovals; (5) indoor and outdoor urban/built areas.	Survey: rating the personal significance of 16 types of settings in the local area. Selecting one type in which their favorite place is represented, and describing that place. Descriptions used to categorize place types.	No	No	Residents of Helsinki and Tampere, Finland. Aged 15–75 years
Korpela and Ylén, 2009	Natural sites favorite places of majority of respondents. Looked at consistency of re-selecting same type of favorite place over a 10-month period. Most frequently selected favorite places in both surveys were small-scale natural state areas, beaches and harbor areas, and large forest areas.	Survey: rating the personal significance of 16 types of settings in the local area. Selecting one type in which their favorite place is represented, and describing that place. Descriptions used to categorize place types.	No	No	Residents of Helsinki and Tampere, Finland. Aged 15-75 years.
Korpela et al., 2010	Natural sites favorite places of majority of respondents. Sixteen favorite place types were grouped into five main place types. Ranked according to frequency: (1) extensively managed natural areas, e.g. woods, forests, meadows; (2) built-up green spaces, e.g. parks; (3) waterside environments, e.g. beaches and harbors; (4) exercise and activity/hobby areas, e.g. playgrounds and sports ovals; (5) indoor and outdoor urban/built areas.	Survey: rating the personal significance of 16 types of settings in the local area. Selecting one type in which their favorite place is represented, and describing that place. Descriptions used to categorize place types.	No	No	Residents of Helsinki and Tampere, Finland. Aged 15–75 years

In general, natural settings were the most commonly identified favorite places, with two exceptions (Korpela, 1991, 1992). Overall, there was great variation in the types of natural environments reported as favorite places, which may be a result of the classification systems used in different studies. Only two studies (Korpela, 1989; Korpela and Hartig, 1996) explored the attributes of favorite places. As shown in Table 1, “beautiful views” and “sunlight” were the most frequently mentioned attributes of favorite places in Korpela and Hartig (1996), and in Korpela (1989) responses such as “homelike” and “peaceful” featured frequently. Two studies also related responses back to the components of restorative environments by using the Perceived Restorativeness Scale (PRS) (Korpela and Hartig, 1996; Korpela et al., 2001). Interestingly, in both of these studies, fascination was found to be the least important component of restoration in favorite places.

### The importance of healthy natural environments

Fascination is a central component of restorative experiences (Kaplan, 1995), and the likelihood of restoration is thought to be greater in natural environments that exhibit more fascinating qualities (Nordh et al., 2009). Fascination has been related to concepts such as naturalness (Nordh et al., 2009; Van Den Berg et al., 2014) and wildness (Annerstedt et al., 2012), which may also relate to ecological quality (Winter, 2012). As fascination refers in part to the ability of an environment to capture and hold one's attention, and natural scenes are considered to “contain many more fascinating features or elements than urban environments” (Joye et al., 2013, p. 3), it stands that dynamic environments containing a greater variety of plant and animal species might thus generate greater fascination. Indeed, visitors to high biodiversity environments have been found to derive a greater level of psychological benefit than visitors to low biodiversity environments (Fuller et al., 2007; Carrus et al., 2015), although this was not directly linked to fascination by the researchers.

There have been repeated calls for research that provides insight into the specific features of natural environments that are required for the attainment of psychological benefits (e.g., Frumkin, 2003; Velarde et al., 2007; Bratman et al., 2012; Keniger et al., 2013). There is a dearth of practical information on this topic available to park agencies tasked with balancing human and environmental benefits in parks and protected places. Furthermore, studies into the health benefits of natural environments have been almost exclusively anthropocentric in nature, and have paid little attention to the health of ecosystems (Jorgensen and Gobster, 2010; Lang and Rayner, 2012). As environmental health and mental health in many urban areas continues to deteriorate, research that prioritizes the health of both human beings and the natural environment will be of increasing importance (Parks Victoria, 2015). As exemplars of restorative, health-giving environments, we believe investigating perceptions of “favorite places” and the natural micro features that people use to explain their connection to these settings will provide valuable information for urban planners seeking to optimize the health benefits of nature. Using previous restoration research as a means to classify responses, we also sought to explore how Australians compared to their European and North American counterparts, in terms of the relative importance they placed on the four components of restorative environments according to ART. The three questions that guided our inquiry were:

1. Which types of natural environments do South Australians value most highly?
2. What attributes of natural “favorite places” do individuals take notice of and use to explain their connection to these places?
3. Does the relative importance of the four ART components in Australian “favorite places” reflect those of European and North American favorite places?

## Methods

### Study setting

The study was conducted in South Australia, a state that spans an area of 984,377 km2 and contains a population of 1.7 million people (Australian Bureau of Statistics, 2015). The state's diverse landscapes, varying described as varying “from rugged outback wilderness and desert to scenic mountain ranges and a coastline that stretches more than 3,700 km” (South Australian Government, 2014, p. 1), made it an ideal location to examine the attributes that are associated with favorite places. The state's population resides primarily in the capital city, Adelaide (77%), but also in large regional centers, and hundreds of small country towns. The study surveyed residents across the State as a whole, to learn more about “favorite places” within South Australia.

### Data collection and procedure

Data collection took place between 1 September and 30 November 2014, using an online questionnaire. The questionnaire consisted of 19 questions exploring participants' use of public and private green spaces, their memories of interacting with nature as children, the centrality of nature in their lives today and a set of demographic questions. The questionnaire also contained two open-ended questions, which form the focus of the present paper. Respondents were asked “what are your favorite outdoor places in South Australia?” and “What is it that you love most about these places?”

Following approval by the University of South Australia Human Research Ethics Committee, the survey was launched on a specially designed webpage that included short human interest stories about outdoor experiences, as well as podcasts of a 6-week radio program titled “Operation Outdoors.” The survey was kept open for 6 weeks after the end of the radio program. The webpage was hosted by ABC Adelaide, who in 2015 held a 12.5% share of South Australia's radio audience; the second most popular radio station in the State (Commercial Radio Australia, 2015). As part of the Australian Broadcasting Commission (ABC), ABC Adelaide's existing website received high traffic and we were confident the popularity of the company's website would lead some people to the Operation Outdoors page independent of promotion. In addition, the page was promoted during the 6-week program of bi-weekly “talkback radio” sessions hosted by two of the authors. The radio sessions focused on an eclectic mix of topics related to the natural environment. Broadly speaking, the radio segments were conversations encouraging people to reminisce about outdoor experiences and the value of those experiences, rather than conversations about specific places. Half of the sessions discussed historical perspectives of green spaces, and included topics such as “A Spring in My Step,” “The Value of Parklands,” and “Drunkenness or Civilization: the Story Behind our Gardens.” This novel method of recruitment was trialed due to the increasing difficulty of engaging the public to complete surveys. Given the indirect method of participant recruitment for this convenience sample, a traditional study response rate cannot be calculated. Participation in the study was voluntary, and no incentive was provided to respondents.

To investigate potential bias, we examined the timing of questionnaire completions, and while there was a peak in responses on the day of each radio session, there was steady traffic throughout the week, and also during the 6 weeks after the program had finished. Audio recordings of each radio session were transcribed, allowing us to examine potential bias in self-reported “favorite places” that might be associated with mentioning specific natural sites during the radio segments. Notably, in the week the radio hosts discussed the history of gardens there were more mentions of the Botanic Garden (17.5% greater that week), compared to the other 11 weeks. This should be taken into consideration when viewing the results.

### Overview of respondents

In total, 447 people completed the questionnaire. The majority of respondents were female (65.7%), mature aged (range: 14–81 years; mean 52 years), and highly educated (53.66% with a bachelor degree or higher), as shown in Table 2. Commensurate with this, the majority of respondent households contained mature/older adults with no children (55.3%, i.e., mature singles and older couples with no children at home). According to census data (Australian Bureau of Statistics, 2015) the demographics of the study sample are not necessarily reflective of the South Australian population, which has a lower proportion of females (50.7%); a younger median age of 39 years; a greater number of households with children (57.8%); and far fewer people with university degrees (14.4%). This is likely a result of the methods used to recruit participants, as both the radio station, and the particular program which included discussions and interviews about the environment and our historic use of it, were more likely to attract an older, more highly educated audience.

**Table 2:** Overview of respondent characteristics (*N* = 447).

**Demographic variable**	**Option**	**Percentage**
Gender	Female	65.7
	Male	34.3
Education	Bachelor degree	30.49
	Postgraduate degree	23.17
	Some undergraduate tertiary	14.02
	Secondary school	13.72
	Vocational/technical training	12.50
	Primary/some secondary school	6.10
Lifecycle	Older couple, no children at home	34.0
	Mature single	21.3
	Middle family (youngest child 6-15 years of age)	12.3
	Mature family (all children over 15 years of age)	10.2
	Young single	9.3
	Young family (youngest child <6 years of age)	7.7
	Young couple, no children	5.2

### Analysis

Participants' favorite places and demographic data were analyzed using SPSS® software to calculate descriptive statistics such as frequencies and means. Participants' responses about why they loved particular places were coded in QSR Nvivo® using inductive content analysis. Directed content analysis was used to examine the relative importance of the four ART components in Australian “favorite places.” The two procedures are explained in the following sections. Similar to Irvine et al.'s (2013) study, where multiple answers were provided by respondents, they were treated as separate, individual statements.

#### Popular types of natural favorite places

Favorite places were initially grouped by name so that we could ascertain which favorite places were shared amongst multiple respondents. This process resulted in 241 unique locations across South Australia. These favorite places were then classified using a modified National Recreation and Parks Association (NRPA) park typology (Mertes and Hall, 1996) to identify the types of natural environments that are favored by the public. The NRPA classification system primarily differentiates parks according to their size, location, and use. However, given the non-spatial method of data collection in the present study, we made several modifications to the park typology that we considered to be more locally indicative of how the parks were used. These changes—such as combining neighborhood, mini, and community parks—are shown in Table 3, which outlines the eight classifications used in the study and provides a rationale for changes made to the original NRPA typology.

**Table 3:** The modified NRPA park typology used to classify “favorite places.”

**Classifications used in this study**	**Description**	**NRPA classifications**	**NRPA size and location guidelines**	**Modification rationale**
Community park	Variable size and location. Recreational green spaces, not dedicated solely to conservation or sports	Community park	Usually between 30 and 50 acres, $\frac{1}{2}$ mile to 3 mile distance	Incorporated neighborhood, mini-parks and large urban parks based on their similarities in intended use, as places of passive and active recreation.
School park	School-owned green spaces, not always publically accessible. Variable size, location determined by school	School park	Variable size, location determined by school	No change.
Sports park	Sports complexes and ovals, location and size variable	Special use, Sports complex	Special use—size variable, location variable	The study sites were specifically sports fields.
			Sports complex—usually a minimum of 25 acres with 40–80 acres optimal, strategically located	
Nature park	Natural resource areas, for example National Parks—size variable, location depends on availability and opportunity	Natural resource areas	Size variable, location depends on availability and opportunity	The new terminology is to clarify that these areas are parks.
Linear park	Greenways and trails—location variable	Park trails, Connector trails	0.5 miles per 1,000 (1983 NRPA standard), location variable	More precise terminology has been used because the connector trails in this study were linear parks.
Botanical gardens and arboreta	Formal botanical gardens, zoos, and arboreta dedicated to the display and study of different species	N/A	N/A	This classification was added to differentiate these green spaces based on their intended use as places of recreation, learning, and the public display of species.
Beach or coastal park	Beaches—Size variable, location depends on availability and opportunity	N/A	N/A	Beaches were differentiated from other natural parks, based on their distinct natural features.
Private green space	Privately owned gardens and back yards	N/A	N/A	Although not a type of park, private green space represented a substantial number of favorite places and warranted differentiation from public parks.
		Neighborhood park	5–10 acres optimal, $\frac{1}{4}$ to $\frac{1}{2}$ mile distance	Neighborhood parks were classified as community parks because they are both managed by local councils, and tend to have more similarities than differences in terms of services, facilities, and use patterns.
		Mini-park	Between 2,500 sq. ft. and one acre, <¼ mile in residential setting	Mini-parks were classified as community parks because they are both managed by local councils, and tend to have more similarities than differences in terms of services, facilities, and use patterns.
		Large urban park	Usually a minimum of 50 acres with 75 or more acres optimal, usually serves entire community	Large urban parks were classified as community parks because they are both managed by local councils, and tend to have more similarities than differences in terms of services, facilities, and use patterns.

#### Loved attributes of natural favorite places

To identify the loved attributes of respondents' favorite places we used an inductive approach to content analysis, whereby we avoided using preconceived categories and instead allowed the categories and their names to flow from the data as we explored it (Hsieh and Shannon, 2005). Although the majority of characteristics identified by Korpela (1989) and Korpela and Hartig (1996) were also in our final word lists, we did not confine our content analysis to the items used in those studies, for several reasons: (1) Lack of background knowledge as to how Korpela and Hartig (1996) derived the initial list of 16 attributes that they provided their respondents with; (2) We believed a list of 16 attributes was unlikely to be sufficiently exhaustive to accurately reflect the experiences of hundreds of respondents in hundreds of different locations; (3) Some of the items used in the previous studies were not suitable for a South Australian context, such as “lake ice” (Korpela and Hartig, 1996); (4) Korpela's (1989) focus was on feelings created by favorite places, and as a result he paid little attention to the attributes he identified as being important, such as “greyness” and “ugliness,” but rather the feelings they conjured; and lastly, (5) There was little consistency between the two previous studies in terms of the attributes they identified, which further encouraged us to err on the side of caution and follow an inductive approach. Whilst conducting the inductive content analysis we believed there was some risk of bias, in that we might misinterpret certain statements simply by restricting them to a single node. To minimize this potential bias, the researchers undertook the first step together, and when necessary, allowed responses to be coded into multiple nodes to avoid making assumptions about intended meanings.

An initial sample of 100 responses was read by the researchers, who agreed there were three broad themes running through the data, namely: descriptions of the physical environment, recreational activities that occurred in favorite places, and the benefits people derived or desired from them. Following this early analysis, we used Moore and Driver's (2005) synthesis of benefit research to strengthen category formation, and using the complete set of responses, highlighted all occurrences in which we believed the respondent was referring to an aspect of the biophysical setting, an activity, or a benefit. Discrepancies were overcome through discussion and the establishment of rules that enabled similar phrases or words to be categorized consistently. Following the extraction of all setting-, activity-, and benefit-related responses, a similar approach using multiple researchers to triangulate results was used in further analysis and coding into sub-nodes. The nodes used in the study are shown in Table 4, along with examples of sub-nodes and participant responses. A complete list of sub-nodes can be obtained from the authors upon request.

**Table 4:** The nodes used in inductive content analysis during the study.

**Domain**	**Nodes**	**Example sub-nodes**	**Example responses**
Setting attributes	Natural attributes	Birds Other fauna, wildlife Plants, vegetation, flora Flowers, orchids, blossoms Aesthetics, beauty, views Quiet, peace, tranquillity, silence Natural processes, seasonal changes Biodiversity and diversity Creeks, rivers, lakes Beach, ocean, sea Sounds and smells of nature Rocks, cliffs, soils, geology Mountains	“Tall trees attracting native birds, hearing and watching bird activities… Seeing the buds burst into color, smelling the flowering creepers and plants, nature's perfume, habitat for insects and butterflies…” ^†^ (Natural attributes; low intensity activities) “Birds and plants are always interesting.” “It's full of native critters; creek systems, caves, waterfalls, billabongs, cliffs, beaches…”
			“The cliffs and hills are a myriad of colors and the views from the top are fantastic. I regularly watch many species of birds including kestrels, peregrine falcons, white-breasted sea eagles, pacific gulls, cormorants, terns, hooded plovers, etc. Brown snakes, lizards, dolphins, NZ fur seals and many other critters, both indigenous and (sadly) exotic.” ^†^ (Natural attributes; low intensity activities).
			“I enjoy…the geology, the wildlife, the creeks and rivers. Any weather and season there is always something new.”
			“…take in the sights, smells and sounds - it is a very sensory experience for me.”
	Human-managed attributes	Accessibility and proximity Picnic and BBQ facilities Swimming pools Seating Art and sculptures Toilets Walking and cycling trails Park maintenance, cleanliness Playground or play space	“Close to home, paths allowing easy access.” “Lots of hiking tracks to walk. Facilities like BBQs…” ^†^ (Human-managed attributes; moderate intensity activities) “A mixture of special plants, garden art and sculptures…” ^†^ (Human-managed attributes; natural attributes) “The athletics field is well maintained.”
			“There are places I like to have coffee, toilets, children's playground, dog poo bags, barbecues, anything you might desire.” ^†^ (Human-managed attributes; low intensity activities).
			“Kept tidy and clean, convenient, traffic is minimal and slow…”
Activities	Low intensity activities	Bird watching Picnics/dining Sitting Fishing Reading	“I can watch the birds eating insects and nectar, scratching in the dirt and collecting material to build nests.” ^†^ (Low intensity activities; natural attributes).
			“A good place to read books and eat a picnic lunch…”
			“…Sitting in the sun, reading quietly, relaxing and feeling the grass beneath my feet.” ^†^ (Low intensity activities; natural attributes, personal benefits).
	Moderate intensity activities	Walking Gardening Cycling Swimming Kayaking	“Hiking, geocaching, kayaking…” “I ride my bike from Paradise to the sea - such fun on the downhill run, though a lift home helps.”
			“I love getting my hands in the dirt and gardening”
			“I love going there for a swim on a hot summer's day.”
	High intensity activities	Sport Running/jogging Mountain Biking	“The ability to have a run around or a kick of the footy.”
			“Good training grounds for running.”
			“Fantastic mountain bike ride up to Cleland…”
Benefits	Personal benefits	Solitude, privacy, escape crowds or city Discovery, exploration, learning Rest and relaxation Improve mood or happiness Spiritual or personal values, connection Nostalgia and memories Physical fitness and exercise Independence, freedom, autonomy Feeling Safe Awe, wonder, marvel	“A chance to escape from a busy and scheduled day-to-day life without phones and screens.” “A sense of being outside the city, personal restoration, exercise.” “Still so much to be explored and discovered…” “…The freedom to explore different footpaths and get lost but always feel safe.” ^†^ (Personal benefits; human-managed attributes).
			“Flinders is a spiritual home - wild, silent, magnificent. I connect with God and the traditional owners.” ^†^ (Personal benefits; social benefits; natural attributes).
			“…Strong childhood memories, so will always love.”
			“…Sense of awe and wonder they engender. Supports spiritual development… De-stress… Re-energise…Development of wisdom through just being there.”
	Social benefits	Family bonding Be with friends Neighborhood relations Teaching, leading, sharing skills	“…Many afternoon teas shared there with family and friends.” ^†^ (Social benefits; low intensity activities).
			“I more easily chat to neighbors if I am in the garden trimming or weeding, so neighborly relations develop naturally.” ^†^ (Social benefits; moderate intensity activities).
			“Teaching my kids about nature and instilling in them an appreciation and respect for nature.” ^†^ (Social benefits; environmental benefits)
	Environmental benefits	Environmental stewardship	“We feel ourselves to be stewards of this land and the ones to look after it…”
			“I am a bush Carer with Trees For Life there. This means that I have a great emotional attachment to it, what I do by planting trees and removing introduced weeds from it greatly helps local native plant species return.” ^†^ (Environmental benefits; personal benefits; natural attributes).
			“Being part of preserving the biodiversity of this area is a huge buzz. Saving the flora and fauna for future generations is very satisfying…” ^†^ (Environmental benefits; natural attributes).

† *Denotes an item that was coded into multiple nodes. The nodes are provided in parentheses following the quote*.

#### Relative importance of ART components in natural favorite places

The third objective of the study was to explore how participants' personal descriptions of their favorite places related to the four components of restorative environments according to ART, i.e., being away, fascination, extent, and compatibility. To do this, we used a directed approach to content analysis, where existing research about restorative environments helped to determine the initial coding scheme (Hsieh and Shannon, 2005). To begin, we compiled lists of words that have been used in previous restorative environments studies to describe the four components of ART (e.g., Kaplan, 1995). Many words and phrases were adopted from the PRS developed by Hartig et al. (1997). After an initial read-through of the responses, we were able to add words and phrases to the lists, which we felt were reflective of particular ART components. At times we used a thesaurus to identify related words, or in the case of “chaos” from the PRS, to identify antonyms. The use of a thesaurus also helped the researchers to reach consensus about which component of ART particular words related to.

As with the inductive coding used to identify loved attributes of favorite places, when necessary, we again allowed responses to be coded into multiple nodes. In doing so, we acknowledged that some responses, such as particular environmental attributes, might be correlated with multiple items. For example, using the PRS, Scopelliti et al. (2012) found that biodiversity was correlated with being away, compatibility, extent, and fascination. It is generally agreed that an interest in observing natural elements is consistent with the construct of fascination, rather than compatibility [e.g., “many interesting things” and “looking at the surroundings” from the PRS (Hartig et al., 1997) and “living things” from Joye et al. (2013)—see Table 5]. Thus, it seems that a significant relation between biodiversity and all four ART components, e.g., in Scopelliti et al. (2012), could be due to a method bias associated with the PRS as discussed by Joye et al. (2013, p. 2)—i.e. correlations between items may simply be “due to employing one common method of measurement for all these items.” Therefore in the present study, unless respondents explicitly referred to engaging in an activity dependent on particular natural features, such as “bird watching,” references to natural elements (including biodiversity) were coded solely as fascination. The word lists are provided in Table 5.

**Table 5:** Word lists used in directed content analysis.

**Component**	**Associated words, phrases and concepts**	**Source**	**Example responses**
Being away	Being away Getting away Distance from: daily hassles, work, routines, ordinary aspects of life Escape unwanted distractions; an escape experience Movement to another setting or another situation Geographical or psychological distance Having a break; taking a break Rest and relaxation Private; privacy Getting away from: the city, traffic, noise Getting outside, getting some fresh air Enjoy the peace and quiet	Kaplan and Kaplan, 1989 Kaplan, 1995 Hartig et al., 1997 Hartig et al., 1997 Hartig et al., 1997 Hartig et al., 1997 Hartig et al., 1997 Authors Authors Authors Authors Authors Authors	“Vast natural spaces that seem far away from man made structures… Far away from traffic and modern life…” ^†^ (Being away; extent). “…Once you are there it feels like “getting away” from city life just for a few hours.” “Being able to switch off from the regular stresses of life.” “Being outside away from computers, lights, advertising… Peace and quiet.”
Fascination	Fascination Beauty Exploration Fascinating qualities Many interesting things Getting to know the place better Explore and discover Looking at the surroundings Living things Changing colors, seasonal changes Constant change, e.g. clouds, flowing rivers Variety of plants and animals Sounds and smells Plants, animals, natural features	Kaplan and Kaplan, 1989 Korpela et al., 2001 Hartig et al., 1997 Hartig et al., 1997 Hartig et al., 1997 Hartig et al., 1997 Hartig et al., 1997 Hartig et al., 1997 Joye et al., 2013 Joye et al., 2013 Joye et al., 2013 Authors Authors Authors	“I enjoy watching the plants and flowers grow. I also enjoy watching the wildlife about the place (lizards, butterflies, spiders etc.). Just going bird watching, the birds are glorious.” ^†^ (Fascination; compatibility). “…I have a feeling of calm and 'slowing down' when I'm observing small things close up - like insects or flower parts or the behaviour of birds - it's these times when I can focus on the now without other thoughts crowding in.” ^†^ (Fascination; being away). “All are home to a myriad of birds, animals and little creatures…” “…I also enjoy the life that crops up boldly by itself - like the lichens on the footpaths and roofs, the single little grass that lives in a tiny hole in the footpath, the small bluetongue poking its head out of an unruly garden, the native cockroaches quietly going about their business in the creeper in a tucked-away local park…” “…The difference in the water whenever one looks at it, depending on the weather.” “…The constant change of colour of the paddocks, the blossom and flowers on the trees, the lambing season, the change in the appearance of sheep after shearing, the noise of the guinea fowl, geese, chooks, pheasants.” “Mostly the sensory pleasure they bring.”
Extent	Being in a whole other world Provides enough to see, experience, and think about Ability to make sense of the environment Distraction Order, harmony (opposite of ‘chaos’ from PRS) There is a lot going on Large enough to explore Space; wide open spaces; lots of space Big enough to get lost	Kaplan, 1995 Kaplan, 1995 Hartig et al., 1997 Hartig et al., 1997 Hartig et al., 1997 Hartig et al., 1997 Bodin and Hartig, 2003 Authors Authors	“The wide open spaces…” “…Wander in another world…just escape…” ^†^ (Extent; being away). “In every place and every time you see something unique and memorable.” ^†^ (Extent; fascination). “I love these spaces because there is lots of space to do whatever you want…” ^†^ (Extent; compatibility).
Compatibility	Feelings of belonging Match between inclinations/activities and environment I can do things I like here The sense that I belong here Sense of oneness with the setting/the environment Being here suits my personality Enjoy this place Reference to activities: walking, picnicking, etc. Time with family and friends Near where I live; proximity to my home Safety; feel safe here	Korpela et al., 2001 Hartig et al., 1997 Hartig et al., 1997 Hartig et al., 1997 Hartig et al., 1997 Hartig et al., 1997 Hartig et al., 1997 Authors Authors Authors Authors	“The peace that being in this place brings my soul.” “Halbury Parklands: The love of my childhood life. My playground and education. The scrub and me were/are one! At 71 years of age I return to it annually to 'ground' myself, to reconnect with what is most important to me…” “I like the wide open spaces and how you can play soccer and ride a bike in the same place.” ^†^ (Compatibility; extent) “…Port Elliot is a 'homing' place for me as I spent many, many weeks as a child there in the caravan park. Even though I go only rarely now, when I do, I have a calm feel, Rooted is the best description.” “Provides a sense of place and connectedness…”

† *Denotes an item that was coded into multiple nodes. The nodes are provided in parentheses following the quote*.

## Results

### Popular types of natural favorite places

**Figure 1:**
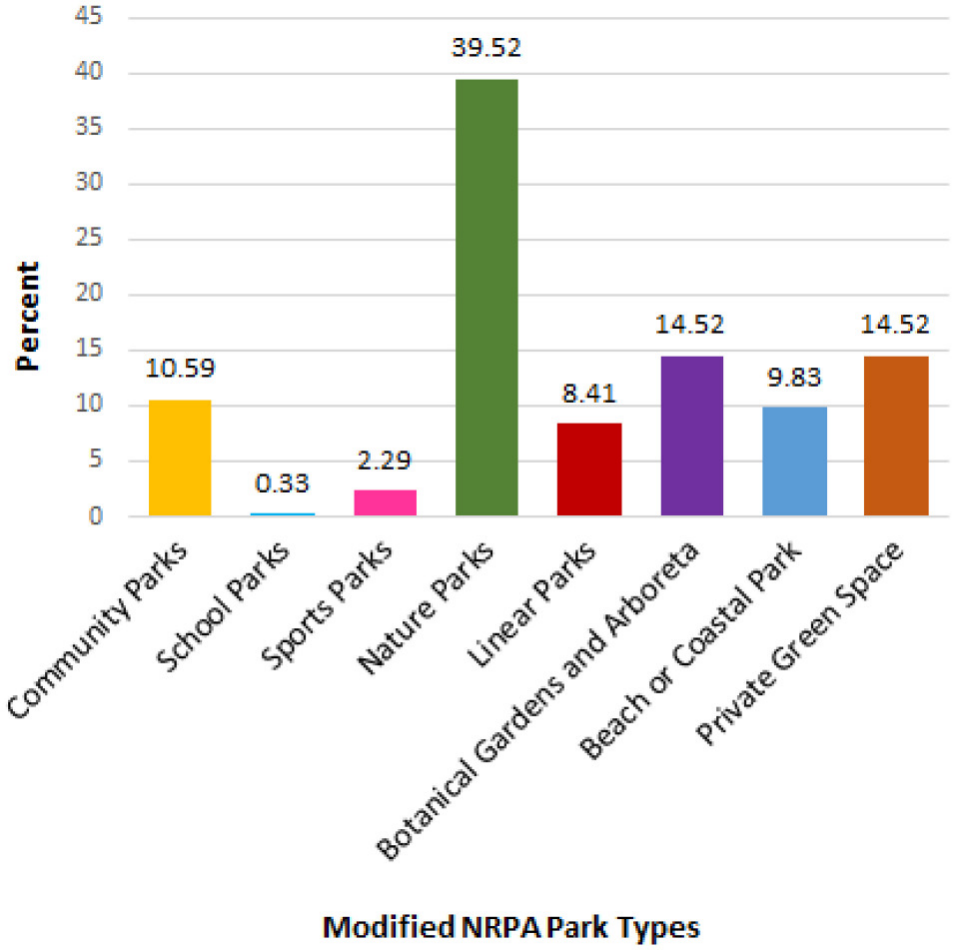
Popularity of different types of green space among participants' self-reported favorite places (*N* = 1022 favorite places).

Respondents were asked the question, “What are your favorite outdoor places in South Australia?” A total of 1,022 favorite places were provided, with respondents generally listing between one and three favorite places. After grouping the favorite places by name, this list was reduced to 241 unique locations. Each unique location was then classified using a modified NRPA park typology (Table 3). Certain responses could not be classified using the typology, as they were either too vague (e.g., “local park”) or referred to a large region that likely contained multiple types of green space (e.g., “the Adelaide Hills”). These responses were coded as “unknown” or “general region”, respectively. The most frequently listed favorite places were “nature parks” such as conservation reserves and National Parks (39.52%) as shown in Figure 1.

The second most frequently listed favorite places were “private green spaces” such as backyards (14.5%) and “botanical gardens and arboreta” (14.5%). The apparent popularity of botanical gardens is particularly interesting, given the number of these green spaces in the State is considerably lower than any other type of green space, i.e., 14.5% of favorite places were comprised of 11 botanical gardens and arboreta, whereas “private green spaces” (also 14.5%) were comprised of hundreds of different gardens and backyards. It should be noted that one of the radio segments promoting the study did discuss one of the State's 11 botanic gardens, however, three botanic gardens featured in the 20 most popular parks in the State (Table 6). The least popular types of favorite places were school parks (0.3%) and sports parks (2.3%).

**Table 6:** The 20 most popular “favorite outdoor places” in the study, ranked by frequency of mention.

**Rank**	**Park name**	**Park type**	**Frequency of mention**	**Distance from CBD^†^ (km)**	**Approximate park size (ha)**
1	Adelaide Botanic Gardens	Botanical garden or arboretum	97	2.3	51
2	Flinders Ranges National Park	Nature park	57	466	93,400
3	Torrens River Linear Park	Linear park or trail	53	1.5	60^*^
4	Belair National Park	Nature park	45	12	835
5	Morialta Conservation Park	Nature park	33	12	533
6	The Adelaide Parklands	Community park	22	2	930
7	Mt Lofty Botanic Gardens	Botanical garden or arboretum	22	19	97
8	Deep Creek Conservation Park	Nature park	15	101	4,496
9	Innes National Park	Nature park	14	288	9,400
10	Cleland Conservation Park	Nature park	11	11	992
11	Onkaparinga River National Park	Nature park	10	33	1,500
12	Mt Lofty Summit	Nature park	10	18	Within Cleland C.P.
13	Coorong National Park	Nature park	9	87	48,990
14	Waterfall Gully	Nature park	9	9.7	608
15	Thorndon Park	Community park	9	11	22
16	Murray River National Park	Nature park	9	75	13,000
17	Wittunga Botanic Gardens	Botanical garden or arboretum	7	15	13
18	Kuitpo Forest	Nature park	6	42	3,600
19	Mt Remarkable National Park	Nature park	6	261	18,270
20	Hazelwood Park	Community park	5	6.4	15

† *Travel distance from center of CBD*.

* *Estimate: Linear park 30 km in length; size calculation based on width of 20 m*.

Excluding private green spaces and ranked according to frequency of mention, the 20 most popular favorite places in South Australia are shown in Table 6. Again, the importance of nature parks is clear, with 13 of the top 20 parks (65%) being comprised of natural areas such as conservation reserves and national parks. Although the frequency with which individual parks were mentioned might seem quite low, we must keep in mind that respondents were surveyed across a State that is almost one million square kilometers in size, contains 352 protected areas, and thousands of community parks. A total of 187 of the 241 places listed were only mentioned by one or two people. Results in Table 6 also suggest the importance of access to greenspace, with half of the parks listed being within close proximity to the majority of respondents (within 15 km of the Adelaide Central Business District). The noteworthy characteristic of the other half of parks, is they are very large and comprise diverse environments and multiple recreation opportunity classes.

Interestingly, despite 87% of South Australia being classed as arid (Department for Environment and Heritage, 2007), only two of the top 20 parks (Flinders Ranges National Park and Mount Remarkable National Park) are located in this arid region. Unlike most of the arid-land parks, both of these parks are situated in mountainous/hilly areas, as are many of the top 20 parks. Half of the top 20 parks (parks 4, 5, 7, 8, 10, 11, 12, 14, 17, and 18) are located in the Mount Lofty Ranges, which surround the capital city of Adelaide. Also of interest, despite a coastline of more than 3,700 km, only three parks listed in the top 20 were coastal parks (parks 8, 9, and 13), although an additional five (parks 3, 11, 14, 15, and 16) included some form of blue space (river, lake, or waterfall).

### Loved attributes of natural favorite places

Respondents were asked the open-ended question, “What do you love about your favorite outdoor places?” Respondents were not prompted to refer to the physical attributes of the setting, nor their own experiences, benefits, or memories, and were free to write whatever came to mind when thinking about their favorite places. Our first step was to identify the proportion of responses that referred to a specific attribute of the biophysical setting, a particular personal benefit, or an activity. Where multiple responses were provided, these were treated as separate statements. Statements that did not fit within a single node were coded into multiple nodes. This initial coding process resulted in 2,460 coded responses. The top 20 “loved” elements of respondents' favorite places are shown in Table 7. Fifteen of the top 20 elements were classified as “setting attributes.” Overall, the most loved attributes of favorite places were birds and plants, which were mentioned with near equal frequency. For example: “The thing that makes it most special is the animal life in the area like native wild birds…” and, “I love watching the Australian native plants grow and attract bees and butterflies and birds…”

**Table 7:** The top 20 “loved” elements of respondents' favorite outdoor places, ranked by frequency of mention.

**Rank**	**“Loved” elements**	**Type**	**Frequency of mention**
1	Birds	Setting attribute	139
2	Plants, vegetation	Setting attribute	137
3	Aesthetics, beauty	Setting attribute	119
4	Wildlife, animals, fauna	Setting attribute	96
5	Walking	Activity	93
6	Nativeness (of species present)	Setting attribute	85
7	Solitude, privacy, escape crowds/city	Benefit	85
8	Quiet, peace, tranquillity, silence	Setting attribute	81
9	Open space, space, vastness	Setting attribute	76
10	Accessibility and proximity	Setting attribute	71
11	Natural processes, seasonal changes	Setting attribute	63
12	Biodiversity and diversity	Setting attribute	54
13	Discovery, exploration, learning	Benefit	49
14	Fresh air, breeze	Setting attribute	45
15	Creeks, rivers, lakes, waterfalls	Setting attribute	45
16	Naturalness, wildness	Setting attribute	45
17	Rest and relaxation	Benefit	43
18	Beach, ocean, sea	Setting attribute	41
19	Family relations	Benefit	38
20	Sounds and smells of nature	Setting attribute	38

Aesthetics was also mentioned with a high degree of frequency, consistent with findings by Korpela and Hartig (1996), e.g., in explaining why they love their favorite place, one respondent wrote: “It is an amazing place of great beauty on the edge of the desert… I greatly appreciate the natural beauty of this place…”

Overall, 64.75% of statements referred to a setting attribute of the favorite place (e.g., “The remnant vegetation and the bird-life to be seen”); 20.7% referred to a personal benefit derived from the place (e.g., “It's a fantastic place for renewing your spirit—escaping the city—and just relaxing”); and 14.5% of statements referred to an activity conducted in the favorite place, e.g., “…a wonderful gift to be able to visit for picnics or tennis or parties.”

Given our interest in providing usable information for park management and nature conservation in Australia, we then identified that 84.6% of loved “setting attributes” referred to natural features of the environment, and 15.4% referred to human-made features such as toilets and walking trails. We further categorized the natural features into elements we believed park managers could modify [such as particular types of plants, e.g., “…I love seeing native plants (groundcovers, heaths, flowering creepers…)” and “…under the shade of beautiful trees, lots of simple things like old logs, mounds to climb on, and play imaginative games. Hard to beat”] and those we considered were beyond reasonable human control (such as the presence of mountains, e.g., “…waterfalls, huge rock-faces and cliffs” and “…breathtaking sandhills and inlets and headlands that seem to never end”). Under this classification system, 70% of statements referring to a “setting attribute” concerned a modifiable natural feature, 15.4% concerned a human-made feature, and 14.6% concerned an unmodifiable natural feature.

In regards to responses lending support to conservation objectives, references to terms such as “biodiversity” and “native species” were surprisingly frequent (e.g., “…great remnant biodiversity,” “It is a desert biodiversity hotspot,” “Tall trees attracting native birds…,” and “I love watching the Australian native plants grow”). Although explicit references to biodiversity were fairly common, there were also many comments about the diversity of plant and animal species written in participants' own words, such as “…many trees of various varieties with different heights, colors and textures” and, “…to see a koala now and then, the kangaroos we've seen on many days, but especially the birds! So many different kinds!” As a result, nativeness, and biodiversity featured in the top 20 “loved” aspects of favorite places.

### Relative importance of ART components in natural favorite places

**Figure 2:**
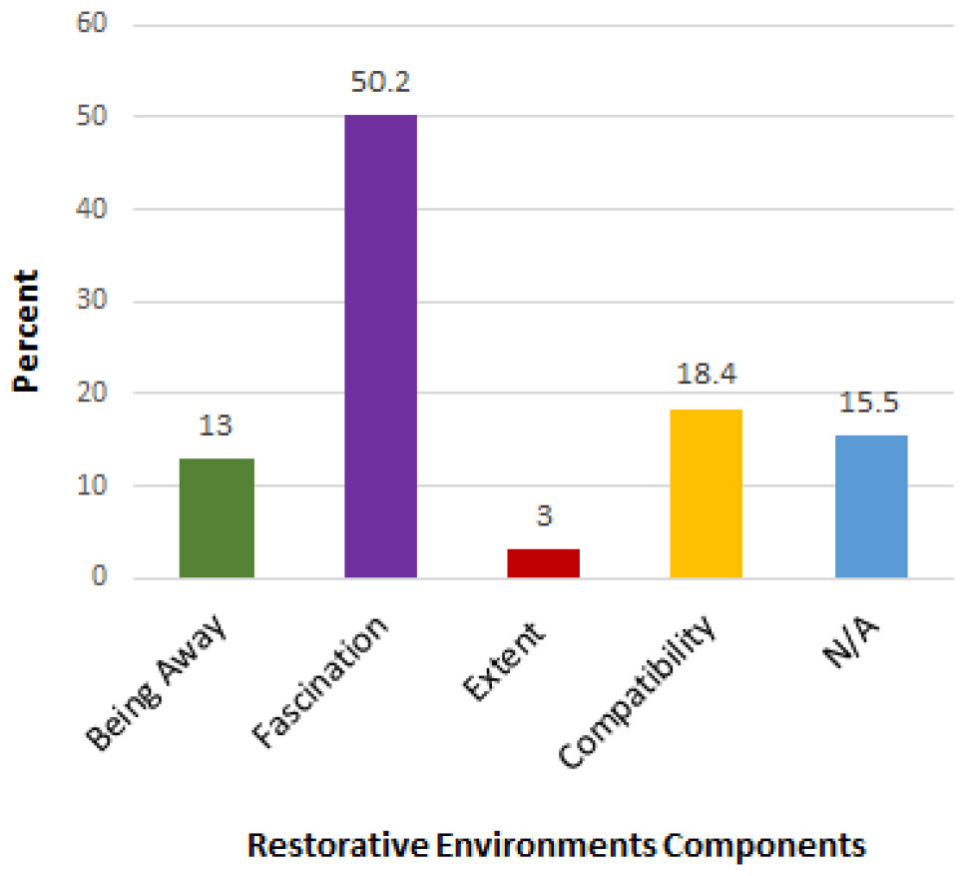
Relative importance of ART components after initial coding, when statements regarding natural features were coded as “fascination.”

To explore the relative importance of the four components of restorative environments in personal descriptions of favorite places, we re-coded responses to the question “What do you love about your favorite outdoor places?” We used a directed approach to content analysis, using words and phrases from previous restorative environments research, including the PRS (Hartig et al., 1997). In contrast to the inclusive and inductive method of content analysis we used when looking at the attributes of favorite places, this time we found that we were more likely to disagree on the category in which certain statements should be placed, particularly in the case of “fascination.” We disagreed on ~5% of cases but after discussion we developed a shared understanding of how we would classify each component. Although we were able to reach a consensus (Table 5) we found that our results differed to those of previous research in this field (Korpela and Hartig, 1996; Korpela et al., 2001), in that fascination was found to be the most important component of favorite places, rather than the least important. This could however, simply be a function of focusing on favorite outdoor spaces, rather than favorite places in general. As shown in Figure 2, more than 50% of statements about what respondents' “loved” about their favorite places were categorized as being indicative of fascination. Extent was found to be the least important component, representing only 3% of responses. We considered 15.5% of responses to not fit within any of the ART categories, e.g., references to food or drink. When specific activities were given as the reason for loving a location, they were coded as “compatibility.” We felt that references to an activity as being something respondents “loved” about their favorite places, were indicative of a match between their inclinations (to conduct that activity) and the suitability of the environment for conducting it in. We coded any reference to a specific natural feature such as orchids, interesting rock formations, or animals as being a sign of fascination, i.e., these features have clearly captured the attention of the respondent, so much so that they have specifically remembered those features when calling their favorite places to mind. We believe such an action was justified, given the Kaplan and Kaplan (1989, pp. 184–185) belief that when discussing “fascinating” stimuli “it would also seem appropriate to include many of the objects found in nature” such as “sunsets and waterfalls, caves, and fires.” Similarly, Kaplan (1995, p. 172) suggests that “fascination can also come from content” such as “wild animals,” and Berto et al. (2010, p. 494) also list “animals, people, water, nature” as fascinating objects. Such features may be sources of fascination because animate or moving objects (like animals and water) capture attention more effectively than static objects (Pratt et al., 2010). Although natural features have consistently been associated with the construct of fascination in previous research (e.g., Kaplan, 1995; Hartig et al., 1997; Joye et al., 2013), responses coded as “fascination” in the present study did not necessarily refer to any attentional outcomes.

## Discussion

### Popular types of natural favorite places

The outdoor “favorite places” of 447 South Australian respondents were classified into eight different green space types using a modified NRPA park typology, similar to that used by Brown et al. (2014). When ranked according to frequency of mention, the types of natural places favored by respondents in our study follows much the same trend to that of Korpela et al.'s (2008) study in Finland. In both studies, the most popular types of favorite places are “nature parks” (or “extensively managed natural areas” such as woods and forests in the Finnish study). Nature parks accounted for nearly 40% of favorite places in the present study. The second most popular place type in the 2008 study was “built-up green spaces” such as parks, which would be equivalent to “community parks,” “botanical gardens,” and “linear parks,” in the present study. Waterside environments such as beaches, followed by exercise areas and sports ovals, are ranked in the same order in both studies. “Private green spaces” and “school grounds” were not included in the Finnish study.

It is reasonable to speculate that people might derive greater restorative benefits from “nature parks,” due to the frequency with which they were identified as being favorite places in the present study, and in Korpela et al. (2008). In this same vein, we might expect to see the most popular “favorite place type” align with the type of green space found to best facilitate psychological outcomes in studies explicitly examining the human benefits of different natural environments. To some degree there is such support, e.g., White et al. (2013) identified forests, coastal areas, and upland areas such as hills and mountains as being the most restorative environments of 16 different place types in England. South Australia is a relatively flat state, however, over 50% of the “top 20 favorite parks” were located in its hilly areas and by far the most frequently mentioned category was “nature parks.” Furthermore, although South Australia is largely arid (Department for Environment and Heritage, 2007), most favorite places were located in the 13% of the state that is not arid, where rainfall is higher, and the presence of forested areas, creeks, and rivers is much greater than in the rest of the state. Barton and Pretty (2010) found that exercising near a beach or river was associated with the greatest improvements in mood and self-esteem, but in Marselle et al. (2013) reductions in post-walk negative affect and perceived stress were associated with farmland and green corridors, but not coastal environments. In contrast to these studies, Marselle et al. (2015) found no significant difference between post-walk affect in different types of green spaces such as nature reserves, urban parks, and farmland. Determining whether these varying—and at times conflicting—results are due to methodological differences between studies, or simply reflect the many nuances of nature-health relationships, requires further research. It is possible that particular types of environments are more effective at facilitating specific psychological benefits, e.g., coastal environments might have a greater effect on restoration, mood, and self-esteem than other environments (Barton and Pretty, 2010; White et al., 2013), but not on negative affect or perceived stress (Marselle et al., 2013).

Marselle et al. (2015) found perceived restorativeness was significantly related to perceived naturalness and perceived biodiversity. Psychological benefits have been found to increase with perceived biodiversity in other studies (e.g., Fuller et al., 2007; Dallimer et al., 2012), however, species diversity is not always accurately detected by respondents. The extent to which participants are able to accurately perceive biodiversity is likely to differ, however, it is assumed that most people are able to distinguish between different types of natural settings, based on the method of self-reporting used to determine “environment type” in many studies (Korpela et al., 2008, 2010; Marselle et al., 2013, 2015; White et al., 2013). Therefore, we can assume that people have some capacity to discern variations in natural attributes, and this has been the case in Fuller et al. (2007) and Johansson et al. (2014) with regard to plant diversity, and in Lamb and Purcell (1990) with regard to naturalness. Greater species diversity and naturalness may be more representative of certain park types in the present study. For example, “nature parks” consisted largely of protected areas, which are known to harbor greater species richness and species abundance than unprotected natural areas (Gray et al., 2016). Furthermore, nature parks are more likely to exhibit the sensory cues (for example Dallimer et al., 2012 suggest that vegetation cover might be an important visual cue) that might influence people's perceptions of biodiversity, naturalness and in turn, restorativeness.

Identifying these sensory cues is of great importance to improving our collective understanding about how people perceive natural environments. At a time when many researchers are arguing that people are becoming increasingly disconnected from the natural world (e.g., Maller et al., 2008), it is valuable to know which aspects of nature people take notice of. It is of particular interest to explore whether people positively perceive features that contribute to the health of natural environments, as opposed to those that contribute only to human activities and experiences. Miller (2005, p. 431) asked, “if people no longer value nature or see it as relevant to their lives, will they be willing to invest in its protection?” Similarly, we might ask, if people no longer take notice of nature in their lives, will they ever come to value it? These questions are beyond the scope of the present study, however, working backwards, we were able to explore the aspects of nature that people use to explain their love for their favorite, and therefore most valued, natural environments.

### Loved attributes of natural favorite places

Writing about one's experiences in nature has been espoused as a form of self-reflection that can improve one's connection with the natural world (Richardson et al., 2015). In the present study, participants were asked to list their favorite outdoor places, and to write about what they love about those places. Participants were not prompted to refer to the features of the environment, nor the benefits or experiences they derive from them. Thus, we believe the results go beyond determining aesthetic and recreation experience preferences, to exploring the transactional relationship between loved environments and the people who value them above all others. We consider that the relationship between person and environment can be mutualistic only if the environment also derives some benefit from being “loved.” We can assume that a person is more likely to protect or advocate for a place that they value, however, it is still of interest to know what it is about valued environments that are important to the people who value them. Developing such an understanding is of particular importance to those designing campaigns aimed at improving nature attachment in disconnected individuals.

When exploring this issue, we first categorized the “loved aspects” of favorite places as referring to a setting attribute, a benefit, or an activity. This process revealed that more than 60% of responses concerned a setting attribute, such as the presence of particular facilities or features of the environment. Of those setting attributes, ~85% referred to natural attributes, rather than artificial or human-created aspects of the environment. Some frequently mentioned attributes were to be expected, such as references to “beauty,” and the proximity of the favorite place to respondents' homes. The beauty of nature has long been considered an important component of human-nature relationships (e.g., Ulrich, 1983), and the proximity, or perceived proximity of parks to people's homes is often a predictor of park use (Giles-Corti et al., 2005; Wang et al., 2015). Interestingly, many responses referred to the micro-variables of natural settings, such as birds, plants, and wildlife. Birds and plants were mentioned with almost equal frequency and overall were the top two “loved” attributes listed by respondents. The importance of plants was not surprising, as plants are almost synonymous with the idea of “nature.” We believe the prominence of birds and wildlife in respondents' writing speaks to the value placed on ecological quality in loved environments. Although some animal species can thrive in low-quality environments, there were often specific references to “native” and “remnant” species, as well as the provision of “habitat.” As suggested by Gobster et al. (2007), the ecological value of an environment might give pleasure to those individuals who are able to recognize it, and this appears to be the case for many of our respondents.

We did not expect many participants to explicitly cite “biodiversity” as a loved feature of their favorite places, given previous research found 60% of respondents had never heard of the term “biodiversity” (Lindemann-Matthies and Bose, 2008). However, a recent study suggests that ecological literacy in South Australia is quite high (Pitman and Daniels, 2016). Perhaps as a reflection of this relatively high level of environmental knowledge in the South Australian population, we discovered that biodiversity was actually frequently mentioned in responses. As a result, species diversity was one of the 20 most frequently mentioned attributes of favorite places. This might be due to the fact that more than 50% of respondents in the study held a bachelor's degree or higher, however, according to Pitman and Daniels (2016, p. 12) education and occupation are not the only factors related to knowledge of the environment, and “ecological literacy need not be the exclusive domain of the highly educated or professionally employed.”

Richardson et al. (2015) sought to identify the positive aspects of “mundane” or “everyday” nature that people took notice of during a 5-day intervention designed to improve nature connectedness. Although respondents in the present study had an existing strong connection with nature, comparing the two studies reveals many similarities in the attributes found to be most important to respondents. The importance of micro-variables is reflected in both studies, with “specific aspects of nature” found to be one of the strongest themes arising from responses in Richardson et al. (2015, p. 613). Participants in both studies similarly identified “beauty,” “wildlife,” “change,” and “sensations” as being important. “Natural processes and seasonal changes” (in the present study) or “growth and temporal changes” in Richardson et al. (2015), were found to be of great importance. Clearly, the ways in which loved environments change throughout the year is noticed by the people who value them, however, the fact that participants in Richardson et al. (2015) noticed change during only a 5 day period, we believe highlights an essential aspect of nature experiences in both “mundane” and “favorite” environments, which is that the living world is never static. Kaplan and Kaplan (1989) suggest that this “ephemera” adds to the perception of fascination and may enhance feelings of “being away.” Beyond this, it would appear that exposure to—and recognition of—dynamic, ever-changing environments can contribute to both the enhancement and maintenance of one's connection with nature. Although the lives of modern people are generally less dependent on the weather and the seasons than those of their ancestors', such variation is still an important and noticeable aspect of their nature experiences. Unlike the increasingly artificial and largely unchanging urban environments that many people now inhabit, the natural world undergoes constant transformation, which is clearly appreciated by many people. It is possible that management actions seeking to improve ecological quality in natural environments should be preceded by interventions that encourage park users to take notice of particular micro-variables and subtle natural processes. By initiating this early engagement, park agencies may find that their actions are received more favorably by a visitor base that has the ability and awareness to perceive the ways such actions simultaneously improve the environment and their enjoyment of it.

### Relative importance of ART components in natural favorite places

According to ART, all four components of restorative environments (“being away,” “fascination,” “extent,” and “compatibility”) are essential to restorative experiences (Kaplan, 1995). Research in Finland and the United States found a significant difference in the apparent importance placed on compatibility and fascination in favorite places, with the latter component found to be of significantly less importance than the former (Korpela and Hartig, 1996; Korpela et al., 2001). As discussed previously, fascination is linked to concepts that may be indicative of ecosystem health, such as species diversity, naturalness, and wildness (Annerstedt et al., 2012; Winter, 2012; Van Den Berg et al., 2014). It has been suggested that “experiencing a favorite place with reference to oneself and one's inclinations appears to be more important than inherently engaging or interesting properties of the environment per se” (Korpela et al., 2001, p. 585). Previous research suggests that actively noticing different aspects of natural environments, such as wildlife and changing foliage colors can improve one's connection with nature (Richardson et al., 2015), and in turn, nature connectedness is related to pro-environmental behaviors (Kals et al., 1999). Given the potential implications of this for conservation outcomes, we sought to explore whether personal descriptions of what people “love” about their favorite places are indicative of a focus on “self” (e.g., “compatibility” between the environment and the activities and benefits desired), or indicative of a focus on the environment (e.g., “fascination” with its interesting or beautiful features).

In the present study, statements associated with the idea of fascination featured prominently in the “loved aspects” of favorite places, particularly those referring to micro-variables such as birds and plants. References to “setting attributes” accounted for 65% of responses, which we believe could be suggestive of a difference between the relative importance of ART components in South Australian favorite places and those in Finland and the United States. The great value placed on fascination in our study may reflect the high level of ecological literacy in South Australia (Pitman and Daniels, 2016), as our survey respondents may be more likely to take notice of and appreciate ecologically valuable, structurally-diverse, species-rich environments. This appreciation may indicate the existence of an “ecological aesthetic” within the sample population (Gobster et al., 2007).

Care should be taken when comparing our results to those of previous research. One of the criticisms of restorative environments research is that most studies have been performed on undergraduate university students in Western Countries (Joye and Van Den Berg, 2013). Studies examining the restorative components of favorite places have similarly focused on students, e.g., the mean participant age across two of Korpela's studies was 23 years (Korpela and Hartig, 1996; Korpela et al., 2001), compared to a mean age in the present study of 52 years. It is possible that people's interest in taking notice of the world around them increases as they age, or conversely, that younger people are more interested in the “self” than older people. It has been suggested that younger people, regardless of their generation, are more narcissistic than their elders (Twenge et al., 2008; Roberts et al., 2010). As “narcissism involves a wide range of self-regulation efforts aimed at enhancing the self” (Twenge et al., 2008, p. 877) and spending time in nature can be thought of as a form of “environmental self-regulation” (Korpela and Ylén, 2007, p. 139), it is possible that the contrasting results between our study and previous studies are age-related. Self-interest aside, it is also possible that the greater importance placed on “compatibility” in previous research is more indicative of “place dependence” rather than “place identity,” based on the traditional, two-dimensional model of place attachment (Williams et al., 1992). Although the two concepts are highly correlated, and both are concerned with a setting that is valued, “place dependence” reflects a functional attachment based on the ability of the valued place to facilitate one's desired experiences, and “place identity,” reflects an emotional or affective bond. It has been suggested that functional attachment may initially draw people to an environment, and that repeated visits, over time, lead to an emotional attachment being formed, i.e., place dependence may precede place identity (Vaske and Kobrin, 2010). Given their mature age, perhaps more respondents in our study have had time to develop stronger emotional connections with their favorite place, and have come to place greater importance on the inherently interesting attributes of the place than on its ability to satisfy their needs.

In the present study, “extent” was found to be the least important component of favorite place experiences. The reason for the apparent difference between our results and those of previous researchers (Korpela and Hartig, 1996; Korpela et al., 2001) may simply be due to the context. South Australians are accustomed to expansive lands and the opportunity to explore them in relatively uncrowded settings. For example, the State's capital city, Adelaide, is located <20 km away from thousands of hectares of conservation land including Belair National Park; and the Central Business District itself is bordered by more than 900 hectares of interconnected parkland. It is possible that “extent” is simply something people take for granted. Likewise, perhaps fascination is part of the national psyche. The Australian national anthem encourages people to take note of the fascinating aspects of the landscape, boasting, “Our land abounds in nature's gifts, of beauty rich and rare.”

### Limitations

Unlike previous research, the present study assumed that experiences of natural favorite places would be restorative, and did not directly measure restorative outcomes or perceived restorativeness. This is a limitation of the study, but we felt it was reasonable to assume most favorite places were indeed restorative environments based on previous research (Korpela et al., 2001). ART itself could also be considered a limitation. While we were interested in examining these relationships, we do concede that the limited sample of previous research may not justify the evolutionary and universalist assumptions underlying the theory (Joye and Van Den Berg, 2013). Lastly, this study is limited by the characteristics of its respondents, who were well-educated older people who clearly value nature. This is an interesting point of difference between our study and previous favorite places research, however, the results should be interpreted with caution. We acknowledge that our respondents' characteristics may be associated with the recruitment method used, as the radio station through which the study was promoted is more likely to attract older listeners. Place attachment researchers examining the effects of age, gender, and education on connections to place have not had consistent results (e.g., discussed in Rollero and De Piccoli, 2010), however, it is highly possible that the types of environments and natural attributes identified as being most important will differ between socio-demographic groups. This study was largely explorative, and we believe further research is needed to improve our collective understanding of how different environmental attributes contribute to restorative outcomes.

## Conclusion

Consistent with European research, the most frequently reported types of favorite places in the present study were “nature parks” such as conservation areas and National Parks. Natural micro-variables such as birds and plants were the most frequently reported “loved” attributes of favorite places, and in general respondents paid much more attention to the physical attributes of their favorite places, rather than their ability to facilitate personal benefits and activities. Accordingly, we found much greater importance was placed on “fascination” in Australian favorite places than in previous research that identified fascination as the least important component of restorative experiences in favorite places. The possible reasons for this contrasting result include the focus on outdoor spaces, the comparably much higher mean age of our respondents, as well as their high level of education. Further, they were sampled from a population likely to have a reasonable knowledge of the natural environment and ecological processes. This is reflected in the personal importance respondents placed on the ecologically valuable attributes of their favorite places, such as the habitat they provide, as well as their species diversity and nativeness. We believe these findings can provide an anchor for marketing strategies aimed at increasing the public's use of parks, and assist in the development of education programs aimed at improving people's understanding of important but intangible concepts such as biodiversity. The findings of this study offer support for interventions that encourage people to take notice of and appreciate nature without overtly seeking to educate them. Beyond exploring *how* we can attract people to nature, we might also ask *why*, i.e., are the attributes of nature that are “lovable” also those that provide health benefits? Further research exploring the ability of different types of environments and environmental features to facilitate psychological benefits, as well as the influence of environmental knowledge on individual perceptions of these environments is warranted. Understanding why people love landscapes is crucial to global efforts to connect people with nature and ultimately improve population health, environmental stewardship and conservation outcomes.

## Ethics statement

This study was carried out in accordance with the recommendations of the Australian Code for the Responsible Conduct of Research, the National Statement on Ethical Conduct in Human Research and the UniSA Framework for the Responsible Conduct of Research. All subjects gave written informed consent in accordance with the Declaration of Helsinki. The protocol was approved by the University of South Australia Human Research Ethics Committee.

## Author contributions

All four authors designed the study and were involved in the collection of data and the coding of responses. Data analyses and interpretation were carried out by MS. The initial and subsequent drafts of the article were written by MS, and critical edits were made by DW, KL, and CD. All authors have approved the paper for publication.

### Conflict of interest statement

The authors declare that the research was conducted in the absence of any commercial or financial relationships that could be construed as a potential conflict of interest.
